# Antimicrobial Stewardship in the Emergency Department Observation Unit: Definition of a New Indicator and Evaluation of Antimicrobial Use and Clinical Outcomes

**DOI:** 10.3390/antibiotics13040356

**Published:** 2024-04-12

**Authors:** Ana Belén Guisado-Gil, Marta Mejías-Trueba, Germán Peñalva, Manuela Aguilar-Guisado, Jose Molina, Adelina Gimeno, Rocío Álvarez-Marín, Julia Praena, Claudio Bueno, José Antonio Lepe, María Victoria Gil-Navarro, José Miguel Cisneros

**Affiliations:** 1 Department of Infectious Diseases, Microbiology and Parasitology, Virgen del Rocío University Hospital, 41013 Seville, Spain; anab.guisado.sspa@juntadeandalucia.es (A.B.G.-G.);; 2 Department of Pharmacy, Virgen del Rocío University Hospital, 41013 Seville, Spain; 3 Institute of Biomedicine of Seville, Virgen del Rocío University Hospital/CSIC/University of Seville, 41013 Seville, Spain; 4 Centro de Investigación Biomédica en Red de Enfermedades Infecciosas (CIBERINFEC), 28029 Madrid, Spain; 5 Emergency Department, Virgen del Rocío University Hospital, 41013 Seville, Spain

**Keywords:** antimicrobial stewardship, Emergency Department, health metrics, antimicrobial agents, bloodstream infection

## Abstract

We aimed to define a novel indicator for monitoring antimicrobial use specifically in the Emergency Department Observation Unit (EDOU) and to assess the long-term impact of an institutional education-based antimicrobial stewardship program (ASP) on the antimicrobial prescribing pattern and clinical outcomes in this setting. A quasi-experimental interrupted time-series study was performed from 2011 to 2022. An educational ASP was implemented at the EDOU in 2015. To estimate changes in antimicrobial use, we designed an indicator adjusted for patients at risk of antimicrobial prescribing: defined daily doses (DDDs) per 100 patients transferred from the Emergency Department to the Observation Unit (TOs) per quarter. The number of bloodstream infections (BSIs) and the crude all-cause 14-day mortality were assessed as clinical outcomes. Antimicrobial use showed a sustained reduction with a trend change of −1.17 DDD per 100 TO and a relative effect of −45.6% (CI95% −64.5 to −26.7), particularly relevant for meropenem and piperacillin-tazobactam, with relative effects of −80.4% (−115.0 to −45.7) and −67.9% (−93.9 to −41.9), respectively. The incidence density of all BSIs increased significantly during the ASP period, with a relative effect of 123.2% (41.3 to 284.7). The mortality rate remained low and stable throughout the study period, with an absolute effect of −0.7% (−16.0 to 14.7). The regular monitoring of antimicrobial use in the EDOU by using this new quantitative indicator was useful to demonstrate that an institutional education-based ASP successfully achieved a long-term reduction in overall antimicrobial use, with a low and steady BSI mortality rate.

## 1. Introduction

Antimicrobial stewardship programs (ASPs) are multidisciplinary initiatives designed to optimize antimicrobial therapy, reduce inappropriate antimicrobial use, improve clinical outcomes and patient safety, and prevent antimicrobial resistance [1]. The benefits of implementing ASPs have been demonstrated in diverse healthcare settings [2,3,4].

Emergency Department Observation Units (EDOUs) are strategic areas where patients receive prompt treatment and are expected to be discharged or admitted to the hospital within a short period of time. There, the diagnosis of infections is very common, and the prescription of antimicrobial agents has a substantial impact on both inpatient and outpatient care [5,6]. Recent guidelines recognize the implementation of ASPs in Emergency Departments (EDs) as a priority [7]. However, it remains a challenge because EDs are characterized by a unique, fast-paced environment with high workloads and patient turnover and a large number of healthcare providers working on shift schedules [8,9].

A further challenge in implementing ASPs in EDs is the lack of indicators to evaluate the effectiveness of these interventions. Although none of the indicators of antimicrobial use are entirely accurate, the measurement of defined daily doses (DDDs) remains a gold standard numerator for comparing drug use data [1]. For inpatients, several denominators have been used to standardize DDDs for hospital censuses, such as occupied bed days, patient days, or admissions [10]. The term “inpatient settings” refers to areas where patients are admitted (e.g., intensive care unit, oncology unit, etc., including procedural areas, such as operating rooms). In contrast, outpatient EDs, pediatric EDs, and 24 h EDOUs are outpatient acute care settings. Thus, the inpatient denominators mentioned above (bed days, patient days, or admissions) cannot be accurately captured electronically from hospital databases for these outpatient acute care settings, and there are no denominators explicitly designed for ED and observation unit counts [11]. Other parameters, such as the number of prescriptions, have been used to evaluate strategy outcomes [12], but standardized indicators for EDs and observation units are needed.

This study aimed to establish a novel indicator for monitoring antimicrobial use in the EDOU and to assess the long-term impact of an institutional education-based ASP on the antimicrobial prescribing pattern and clinical outcomes.

## 2. Results

During the study period, the ED received 1,456,664 visits, excluding pediatrics, trauma patients, and obstetric and gynecologic emergencies. Of these, 238,876 patients, an average of 16%, were transferred to the EDOU, where they stayed for a mean of 21 h (range 18–22 h). Since the inception of the ASP, three training courses consisting of clinical sessions were held in 2015, 2018, and 2019. In addition, 38 reports were written and disseminated, including one per quarter and an additional annual report to the department head, on the level of achievement of pre-agreed objectives.

### 2.1. Antimicrobial Use

**Figure 1:**
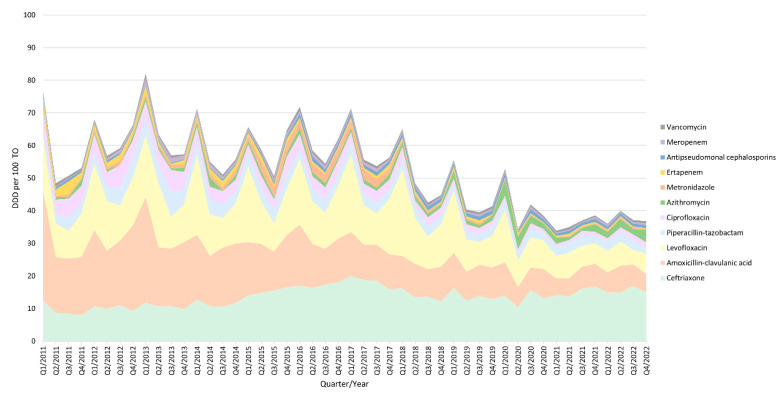
Antimicrobial use during the study period (2011–2022). Data are presented as defined daily doses (DDDs) per 100 patients transferred to the Observation Unit (TOs).

The mean global consumption in the EDOU was 59.4 ± 13.1 DDDs per 100 patients transferred from the ED to the Observation Unit (TOs). Ceftriaxone (13.7 ± 3.0), amoxicillin-clavulanic acid (13.7 ± 6.6), and levofloxacin (12.8 ± 5.4) were the most commonly used antimicrobials. Antimicrobial use was characterized by seasonal variations with peaks in the first quarters of the year (winter), with a seasonal factor of 16% above the average, and troughs in the third quarters of the year (summer), with a seasonal factor of −10%. In the second quarter of 2020, coinciding with the COVID-19 pandemic, this pattern disappeared (Figure 1). The mean antimicrobial use decreased from 68.7 ± 9.4 DDDs per 100 TOs in the pre-intervention period to 53.9 ± 11.9 DDDs per 100 TOs in the ASP period (−21.5%; CI95% −11.8 to −31.1; *p* < 0.001). Detailed pre-post analyses of the mean use per antimicrobial are provided in Table S1 (see online Supplementary Materials).

**Figure 2:**
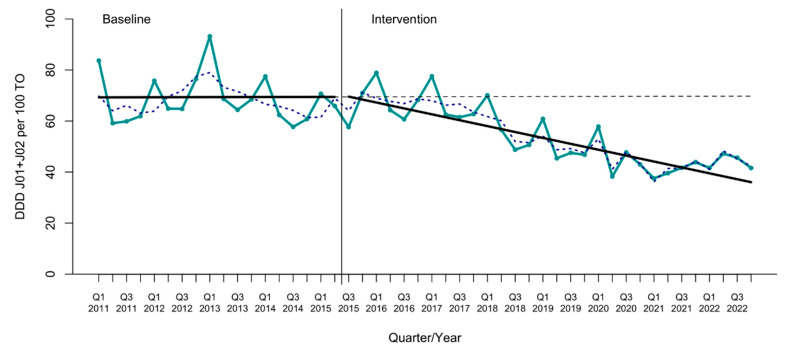
Interrupted time-series analysis of total antimicrobial use (ATC J01 + J02). Solid lines show the observed trend during the pre-intervention and intervention periods. The dashed line shows the expected trend after the intervention according to the pre-intervention values. The dotted blue line shows the deseasonalized series. DDDs, defined daily doses. TOs, patients transferred to the Observation Unit. Q, quarter.

The interrupted time-series analysis (ITSA) showed a sustained reduction in total antimicrobial use since the inception of the ASP, with a change in the trend of −1.17 DDDs per 100 TOs and a relative effect of −45.6% (CI95% −64.5 to −26.7) compared to the expected use at the end of the study (Table 1, Figure 2, and Figures S1–S11, see online Supplementary Materials). Broad-spectrum antimicrobials, such as meropenem and piperacillin-tazobactam, showed large reductions with relative effects of −80.4% (CI95% −115.0 to −45.7) and −67.9% (CI95% −93.9 to −41.9), respectively. Among the antimicrobials commonly used for respiratory infections, the use of levofloxacin (−70.1%, CI95% −83.8 to −56.4) and ceftriaxone (−34.5%, CI95% −67.6 to −1.4) decreased significantly. There was also a reduction in the use of metronidazole (−98.9%, CI95% −116.9 to −80.8).

### 2.2. Clinical Outcomes

The average yearly number of blood cultures collected in the ED was 16 per 100 ED visits (range 13–18), and this number increased over the study period (*p* < 0.0001) (Table S2, see online Supplementary Materials).

#### 2.2.1. Incidence Density

*Escherichia coli* was the most common microorganism causing bloodstream infections (BSIs) (Table S3, see online Supplementary Materials). The percentage of bacteria with some mechanism of microbial resistance for each species was as follows: 9.5% extended-spectrum β-lactamase (ESBL) *E. coli*, 15.3% ESBL *Klebsiella pneumoniae*, 6.0% multidrug-resistant (MDR) *Pseudomonas aeruginosa*, and 11.1% methicillin-resistant *Staphylococcus aureus*. Time-series analyses of the incidence density of BSIs during the study period are shown in Figure S12 (see online Supplementary Materials).

**Figure 3:**
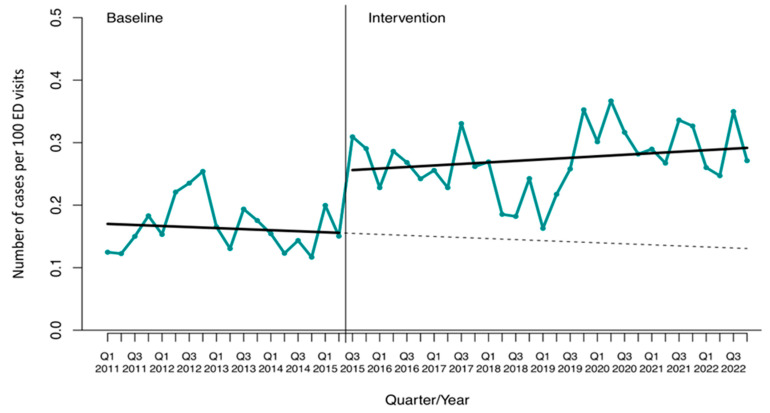
Interrupted time-series analysis of the trends in the incidence density (ID) of overall bloodstream infections (BSIs) observed before and after the implementation of the antimicrobial stewardship program. Solid lines show the observed trend during the pre-intervention and intervention periods. The dashed line shows the expected trend after the intervention according to the pre-intervention values. ED, Emergency Department. Q, quarter.

The pre-post analysis showed an increase in the mean incidence density of overall BSIs (0.17 ± 0.04 vs. 0.27 ± 0.05 cases per 100 ED visits; *p* < 0.001) (Table S4, see online Supplementary Materials). The ITSA found that the overall BSI incidence density level increased significantly during the ASP period, with a relative effect of 123.2% (CI95% 41.3 to 284.7) at the end of the study period, mainly due to non-MDR bacteria (Figure 3 and Table S5, see online Supplementary Materials).

#### 2.2.2. Mortality Rate

The overall mortality rate on day +14 for patients with bacteremia was 9.3%. *P. aeruginosa*, *S. pneumoniae*, and *S. aureus* showed the highest mortality rates, whereas *E. coli* presented the lowest (Table S3, see online Supplementary Materials). Time-series analyses of the mortality of BSIs are depicted in Figure S13 (see online Supplementary Materials).

**Figure 4:**
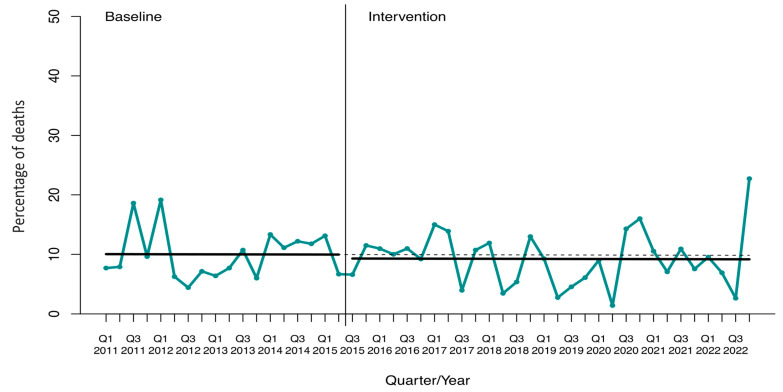
Interrupted time-series analysis of the 14 d mortality rate of overall bloodstream infections. Solid lines show the observed trend during the pre-intervention and intervention periods. The dashed line shows the expected trend after the intervention according to the pre-intervention values. Q, quarter.

There were no significant differences in the mean overall mortality rate of BSIs on day +14 pre- and post-intervention (10 ± 4% vs. 9 ± 5%; *p* = 0.587) (Table S6, see online Supplementary Materials). The ITSA showed that the mortality rate remained low and stable throughout the study period, with an absolute effect of −0.7% (CI95% −16.0 to 14.7) compared to the expected mortality rate at the end of the study (Figure 4 and Table S7, see online Supplementary Materials).

## 3. Discussion

This study presents a new quantitative indicator specifically designed to monitor antimicrobial use in the EDOU and demonstrates that an education-based and institutionally supported ASP in the EDOU was successful in achieving a sustained reduction in overall antimicrobial use and improving the prescription profile, particularly for broad-spectrum antimicrobials targeting Gram-negative bacteria and those commonly prescribed for respiratory infections. The reduction in antimicrobial consumption during the intervention is even more valuable when we consider that, during this period, there was a significant increase in the performance of blood cultures and the incidence density of bacteremia in the ED. The intervention was also safe, with a consistently low mortality rate.

Although our primary objective was not to assess the influence of the emergence of SARS-CoV-2, our research spanned eleven years, the last few of which were substantially affected by the COVID-19 pandemic. Before this period, we observed a seasonal pattern in the use of antimicrobials that was consistent with the trend in outpatient antimicrobial data in previous studies [13]. Seasonality analysis revealed a flattening of this pattern after the onset of the COVID-19 pandemic compared to previous years.

Regarding antimicrobial use monitoring, the proportion of patients receiving antibiotic prescriptions is the most frequently employed indicator according to the results of a previous review focusing on the particular aspects of ASPs in the ED [12]. A limited number of studies used DDDs as the numerator adjusted to 100 patient days [14,15]. “Patient days” is the manual or electronic count of the number of patients at the site measured at the same time each day and, like occupied bed days, has traditionally been used to assess inpatient antimicrobial use. Both denominators may miss part of the potential time a patient could be exposed to antimicrobials, depending on the timing of the daily census, and quite often exclude areas, such as EDs and observation units [16]. Recently, a multidisciplinary group of Spanish experts in the management of infections in EDs and the implementation of ASP evaluated a proposal of indicators using a modified Delphi method. For antimicrobial use, “DDD per 100 patients” was selected as a high-priority indicator [17]. In our study, we established the indicator “DDD per 100 patients transferred from the ED to the Observation Unit” as a specific indicator for EDOUs, which is accurate and easily captured from the hospital database, and we demonstrated that it can be useful for intra-facility and inter-facility comparisons. Thus, “DDD per 100 patients transferred from the ED to the observation unit” could be considered a more precise definition of how “DDD per 100 patients” should be assessed in EDOUs. Assessing the antimicrobial use in our organization using this new indicator and measuring the impact on bacteremic infections are two important aspects that have allowed us to indirectly monitor the appropriateness of antibiotic consumption for serious infections in the community. The selection of appropriate empirical treatment for the type of infection likely to result in hospital admission is crucial for ensuring prompt care, which justifies its prioritization within our ASP.

Comparing the results of our ASP with other stewardship strategies is challenging because of the heterogeneity in the indicators used to monitor antimicrobial use, as well as the fact that most previous experiences with ASP in the ED have been geared towards improving specific infectious syndromes (e.g., urinary tract infections, community-acquired pneumonia, or skin and soft tissue infections, among others), populations (e.g., pediatric patients), or antibiotic classes [18,19]. Borde et al. [14] implemented a multifaceted ASP focused on broad-spectrum cephalosporin and fluoroquinolone use in a medical ED. Their findings indicate a decrease in overall antimicrobial use, primarily attributed to a decline in third-generation cephalosporins. However, this study did not assess clinical or microbiologic outcomes. In contrast, Savoldi et al. [15] evaluated the effects of a non-restrictive ASP in a medical ED and showed a two-thirds reduction in antimicrobial costs, but not in antimicrobial use measured as DDDs per 100 patient days, with no impact on mortality. Both studies were conducted in large German university hospitals, where antimicrobial consumption rates were considerably higher than those observed at our center. One of the findings to highlight in our case is that the use of the new indicator allowed us to quantify the sustained reduction found in the use of broad-spectrum antimicrobials, such as meropenem and piperacillin-tazobactam, after the start of the ASP, which is even more valuable considering that the incidence of MDR bloodstream infections increased during the intervention period.

There is a paucity of ASP studies with outcomes other than antimicrobial use rates. Measures of meaningful clinical and public health outcomes, such as adverse events, patient mortality, and community rates of antimicrobial resistance, are scarce [12]. Considering relevant clinical outcomes, not only the evolution of the incidence of BSIs but the mortality rate of these infections, in addition to robust statistical analyses for longitudinal data using ITSA, are some of the strengths of our work. Concerning clinical outcomes from BSIs, *Enterobacterales* species, especially *E. coli*, were the main species causing bacteremia in the ED, and no carbapenemase-producing isolates were detected in our study. Previous studies in the ED showed similar results regarding *E. coli* and *S. aureus* representativeness; an article designed to evaluate the frequency of the appropriate empirical treatment of *Enterobacterales*-associated BSIs in the ED of a French teaching hospital over 13 years reported *E. coli* as the main species isolated [20]. Likewise, another retrospective analysis of bacterial pathogens in patients with bacteremia presenting to the ED showed that the most common Gram-positive organism was *S. aureus* [21]. Regarding overall BSIs, we observed an increase in the incidence over time that could be explained by the efforts of front-line healthcare professionals to perform high-quality diagnostic microbiological tests before starting empirical therapy, which is in line with the recommendations of antimicrobial stewardship teams [22].

This study has some limitations. First, while the extended analysis period allowed our research team to create a reliable indicator of antimicrobial use for our center, variations in the roles, organization, admission criteria, length of stay, and care provided in other EDOUs around the world make it difficult to draw generalizable conclusions before demonstrating its external validity. Second, there are additional important factors to consider when evaluating antimicrobial use in the ED, such as the indication for oral ambulatory treatment and its duration. These considerations are not adequately addressed by this indicator and are outside the scope of this study. Finally, due to limitations in the hospital records database and software, we were unable to obtain data on infections other than those regularly monitored in the ASP.

## 4. Materials and Methods

### 4.1. Study Design

A quasi-experimental before and after study was conducted using ITSA. In 2011, an education-based and institutionally supported ASP, named PRIOAM, was implemented hospital-wide and has been ongoing since then [23]. Specific antimicrobial stewardship interventions started in the EDOU in the second quarter of 2015. For the ITSA, the study period covered 48 quarters (12 years): from 1 January 2011 to 30 June 2015 (pre-intervention period) and from 1 July 2015 to 31 December 2022 (ASP period).

The study was performed at a 1266-bed tertiary care teaching hospital covering a population of 564,399 in Seville, Andalusia, Spain. The EDOU in our center has 52 beds for the support of circulatory, digestive, and respiratory diseases as the most common pathologies for medical consultations. Obstetric and gynecologic emergencies, the pediatric observation unit (13 beds), and the trauma observation unit (26 beds) were excluded from the study.

The study protocol was approved by the Ethics Committee of the University Hospital Virgen del Rocio (Project ID: PI-0361-2010; 22 December 2010). Considering the risks and potential harms involved in the research, the Ethics Committee authorized the waiver of informed consent.

### 4.2. Intervention

PRIOAM is a set of educational strategies implemented by a multidisciplinary team consisting of infectious disease, microbiology, pharmacy, intensive care, pediatrics, prevention, and nursing professionals. The results obtained by PRIOAM in the whole center and specific settings other than the EDOU have already been published [23,24,25].

For the EDOU, the ASP strategies were focused on the development and updating of clinical guidelines for the diagnosis and treatment of common infectious syndromes (https://www.guiaprioam.com/; accessed on 2 February 2024), clinical sessions to address practical aspects of common infections, the inclusion of ASP goals in the annual agreement signed by the hospital director, and dissemination of quarterly reports on the evaluation of those goals. In addition, the microbiology department reports all patients with positive blood cultures on a 24/7 basis to the infectious disease specialist, who conducts bedside consultations in the EDOU [26].

### 4.3. Outcomes

Due to the lack of a standardized indicator, we designed a specific metric adjusted for patients at risk of antimicrobial prescribing in the EDOU. For the numerator, we chose the DDDs proposed by the World Health Organization (WHO) [27], because the standardization and widespread use of this metric allows for a comparison between antimicrobial agents at local, national, and international levels. For the denominator, we selected the total number of patients transferred from the ED to the Observation Unit (TO) during a given period. The effect of ASP on antimicrobial use was assessed through quarterly measures of DDDs per 100 TOs, globally for antibacterials for systemic use (ATC group J01) and antifungals (ATC group J02), and specifically for those antimicrobials for which mean consumption was >0.5 DDDs per 100 TOs. The DDDs were prospectively collected from the computerized pharmacy database of drugs dispensed to the EDOU. Data on TOs were obtained from the hospital records database.

To monitor the clinical outcomes, the number of BSIs and the crude all-cause mortality rate at day +14 after diagnosis of BSI caused by *E. coli*, *K. pneumoniae*, *P. aeruginosa*, *S. aureus*, or *S. pneumoniae* were recorded among all blood cultures obtained in the ED. These outcomes were monitored quarterly as the number of cases per 100 ED visits (incidence density) and the percentage of deaths (mortality rate), both overall and those caused by MDR bacteria, such as ESBL *E. coli*, ESBL *K. pneumoniae*, MDR *P. aeruginosa*, and methicillin-resistant *S. aureus*. Antibiotic susceptibility was assessed according to the Clinical and Laboratory Standards Institute (CLSI) Guidelines and/or the European Committee on Antimicrobial Susceptibility Testing (EUCAST) criteria [28,29]. For MDR categorization, the German Society for Hygiene and Microbiology criteria were considered [30]. The number of blood cultures collected annually in the ED per 100 ED visits was also measured.

### 4.4. Data Analysis

For the description, categorical variables were expressed as frequencies and proportions, and continuous variables were reported as means ± standard deviations. Seasonality was also evaluated and seasonal factors of antimicrobial use were calculated. Student’s *t*-tests or Mann–Whitney U tests were used for univariate pre-post analyses. Normality was tested using the Kolmogorov–Smirnov test.

To evaluate the ASP’s effects, we conducted a pre-post ITSA to estimate changes in levels and trends after the inception of the program. Generalized least squares regression with autoregressive moving average models was used to account for the autocorrelation in the longitudinal data. Akaike’s information criterion with the validation of autocorrelation structures by likelihood ratio tests [31] was used for the final model selection for each variable. Absolute and relative (%) effects were calculated as the difference between the expected pre-intervention trend and the modeled end-of-study trend to estimate the long-term effect attributable to ASP for each outcome. To avoid confounding factors related to the COVID-19 pandemic, the COVID-19 pandemic was modeled as a dummy variable in the ITSA for antimicrobial use and clinical outcomes.

Confidence intervals [CI95%] or *p*-values were included to indicate statistical significance. Differences were considered statistically significant at *p* < 0.05 (2-tailed tests). IBM SPSS Statistics software v. 23.0 and R software v. 3.5.2 were used for statistical analyses.

## 5. Conclusions

In conclusion, this study presents a new quantitative indicator specifically designed to monitor antimicrobial use in the EDOU. Our results show that the regular monitoring of antimicrobial use in the EDOU using this metric demonstrated that an institutional education-based ASP successfully achieved a long-term reduction in overall antimicrobial use and improved the prescribing profile of certain antimicrobial groups in this setting, with a steadily low BSI mortality rate.

## Figures and Tables

**Table 1:** Interrupted time-series analysis of changes in trends of antimicrobial use.

Outcomes	Regression Intercept	Pre-Intervention Trend	Change in Level ^a^	Change in Trend ^b^	Absolute Effect	Relative Effect ^c^ (%)
Total J01 + J02	69.28 (62.80 to 75.75)	0.01 (−0.55 to 0.57)	1.28 (−4.99 to 7.55)	−1.17 (−1.92 to −0.41)	−31.81 (−54.48 to −9.14)	−45.6 (−64.5 to −26.7)
Ceftriaxone	8.99 (5.83 to 12.17)	0.28 (0.02 to 0.55)	1.07 (−1.58 to 3.71)	−0.27 (−0.63 to 0.09)	−7.79 (−18.64 to 3.05)	−34.5 (−67.6 to −1.4)
Amoxicillin-clavulanic acid	24.22 (22.52 to 25.92)	−0.40 (−0.56 to −0.24)	−2.36 (−4.58 to −0.14)	0.05 (−0.15 to 0.26)	−0.05 (−6.17 to 6.06)	−1.1 (−120.5 to 118.3)
Levofloxacin	12.99 (10.71 to 15.26)	0.19 (0.01 to 0.36)	0.62 (−0.06 to 1.30)	−0.55 (−0.80 to −0.31)	−15.4 (−22.7 to −8.2)	−70.1 (−83.8 to −56.4)
Piperacillin-tazobactam	3.71 (2.47 to 4.95)	0.06 (−0.05 to 0.17)	−0.64 (−1.94 to 0.66)	−0.15 (−0.29 to 0.001)	−4.52 (−8.92 to −0.12)	−67.9 (−93.9 to −41.9)
Ciprofloxacin	6.22 (5.35 to 7.12)	−0.13 (−0.22 to −0.06)	0.43 (−0.55 to 1.41)	0.02 (−0.08 to 0.13)	1.01 (−1.91 to 3.93)	155.2 (−655.8 to 686.8)
Azithromycin	0.66 (0.17 to 1.14)	0.03 (−0.01 to 0.08)	0.26 (−0.37 to 0.89)	−0.03 (−0.09 to 0.02)	0.16 (−2.87 to 3.19)	7.1 (−75.9 to 90.1)
Metronidazole	0.22 (−0.84 to 1.28)	0.11 (0.03 to 0.18)	0.26 (−0.23 to 0.75)	−0.18 (−0.29 to −0.08)	−5.22 (−8.45 to −1.99)	−98.9 (−116.9 to −80.8)
Ertapenem	2.16 (1.93 to 2.39)	−0.06 (−0.08 to −0.04)	0.08 (−0.26 to 0.41)	0.02 (−0.009 to 0.04)	0.14 (−0.47 to 0.68)	14.3 (−19.7 to 47.7)
Antipseudomonal cephalosporins	0.53 (0.35 to 0.71)	0.004 (−0.01 to 0.02)	0.46 (0.36 to 0.56)	−0.006 (−0.02 to 0.01)	0.27 (−0.31 to 0.85)	36.9 (−68.9 to 142.9)
Meropenem	0.54 (0.08 to 1.01)	0.02 (−0.02 to 0.06)	0.17 (−0.26 to 0.60)	−0.06 (−0.11 to −0.006)	−1.16 (−2.71 to 0.40)	−80.4 (−115.0 to −45.7)
Vancomycin	0.74 (0.58 to 0.90)	−0.01 (−0.02 to 0.004)	0.28 (0.07 to 0.49)	−0.004 (−0.02 to 0.01)	0.20 (−0.37 to 0.78)	83.0 (−341.2 to 507.3)

Data are presented as quarterly defined daily doses per 100 patients transferred to the observation ward with a 95% confidence interval unless otherwise specified. J01 refers to the WHO ATC code for antibacterials for systemic use. J02 refers to the WHO ATC code for antimycotics for systemic use. ^a^ Increase or decrease in the first quarter after the start of the antimicrobial stewardship program (ASP) period with respect to the expected value. ^b^ Change in the slope for the ASP period. ^c^ Percentage difference between the expected value according to the pre-intervention trend and the trend at the end of the ASP period. “Antipseudomonal cephalosporins” refer specifically to cefepime and ceftazidime.

## Data Availability

The datasets used and/or analyzed during the current study are not publicly available due to privacy restrictions.

## Supplementary Materials

The following supporting information can be downloaded at: https://www.mdpi.com/article/10.3390/antibiotics13040356/s1. Table S1. Differences between the pre-intervention period and the antimicrobial stewardship program (ASP) period regarding pre-post analysis of antimicrobial use. Figure S1. Interrupted time-series analysis of the trends in ceftriaxone observed before and after the implementation of the antimicrobial stewardship program. Figure S2. Interrupted time-series analysis of the trends in amoxicillin-clavulanic acid observed before and after the implementation of the antimicrobial stewardship program. Figure S3. Interrupted time-series analysis of the trends in levofloxacin observed before and after the implementation of the antimicrobial stewardship program. Figure S4. Interrupted time-series analysis of the trends in piperacillin-tazobactam (PTZ) observed before and after the implementation of the antimicrobial stewardship program. Figure S5. Interrupted time-series analysis of the trends in ciprofloxacin observed before and after the implementation of the antimicrobial stewardship program. Figure S6. Interrupted time-series analysis of the trends in azithromycin observed before and after the implementation of the antimicrobial stewardship program. Figure S7. Interrupted time-series analysis of the trends in metronidazole observed before and after the implementation of the antimicrobial stewardship program. Figure S8. Interrupted time-series analysis of the trends in ertapenem observed before and after the implementation of the antimicrobial stewardship program. Figure S9. Interrupted time-series analysis of the trends in cefepime and ceftazidime observed before and after the implementation of the antimicrobial stewardship program. Figure S10. Interrupted time-series analysis of the trends in meropenem observed before and after the implementation of the antimicrobial stewardship program. Figure S11. Interrupted time-series analysis of the trends in vancomycin observed before and after the implementation of the antimicrobial stewardship program. Table S2. Data of the blood cultures collected annually in the Emergency Department (ED) per 100 ED visits. Table S3. Incidence of bloodstream infections and mortality of patients diagnosed with bloodstream infections by causative microorganisms (2011–2022). Figure S12. Time-series plot of incidence density of bloodstream infections during the study period (2011–2022). Data are presented as the quarterly number of cases per 100 Emergency Department (ED) visits. Table S4. Differences between the pre-intervention period and the antimicrobial stewardship program (ASP) period regarding the pre-post analysis of incidence density of bloodstream infections. Table S5. Interrupted time-series analysis of changes in trends of the incidence density of bloodstream infections. Figure S13. Time-series plot of mortality rate of bloodstream infections during the study period (2011–2022). Data are presented as quarterly percentage of all-cause deaths on day +14. Table S6. Differences between the pre-intervention period and the antimicrobial stewardship program (ASP) period regarding the pre-post analysis of the mortality rate of bloodstream infections. Table S7. Interrupted time-series analysis of changes in trends of the mortality rate of bloodstream infections.
