# Supplementary material for: Antimicrobial Stewardship in the Emergency Department Observation Unit: Definition of a New Indicator and Evaluation of Antimicrobial Use and Clinical Outcomes
Source: Antibiotics (Basel). 2024 Apr 12;13(4):356. doi: 10.3390/antibiotics13040356 (PMC11047618; doi:10.3390/antibiotics13040356)
Supplement: Supplementary file 1 [file antibiotics-13-00356-s001.zip › antibiotics-2897002-supplementary.pdf]

Supplementary Materials

Table S1. Differences between the pre-intervention period and the antimicrobial stewardship program (ASP) period regarding pre-post analysis of antimicrobial use.

**Outcomes**	**Pre-Intervention Period**	**ASP Period**	***p*-value**
Total J01+J02	68.7 ± 9.4	53.9 ± 11.9	<0.001
Ceftriaxone	10.9 ± 1.8	15.3 ± 2.2	<0.001
Amoxicillin-clavulanic acid	20.2 ± 5.4	9.7 ± 3.2	<0.001
Levofloxacin	14.7 ± 4.9	11.7 ± 5.5	0.058
Piperacillin-tazobactam	4.5 ± 1.2	3.0 ± 0.6	<0.001
Ciprofloxacin	4.9 ± 1.1	2.4 ± 1.3	<0.001
Azithromycin	1.1 ± 0.6	1.8 ± 1.0	0.136
Metronidazole	1.0 ± 0.6	1.1 ± 1.1	0.136
Ertapenem	1.7 ± 0.8	0.6 ± 0.3	<0.001
Antipseudomonal cephalosporins	0.5 ± 0.2	1.0 ± 0.2	<0.001
Meropenem	0.8 ± 0.5	0.6 ± 0.3	0.766
Vancomycin	0.6 ± 0.2	0.6 ± 0.2	0.409

Data are presented as the mean ± standard deviation of quarterly defined daily doses (DDDs) per 100 patients transferred to the Observation Unit (TOs).
p-values represent the results from Student's t-tests or Mann-Whitney U tests, according to the data distribution. ASP, antimicrobial stewardship program.
Antipseudomonal cephalosporins refer specifically to cefepime and ceftazidime.

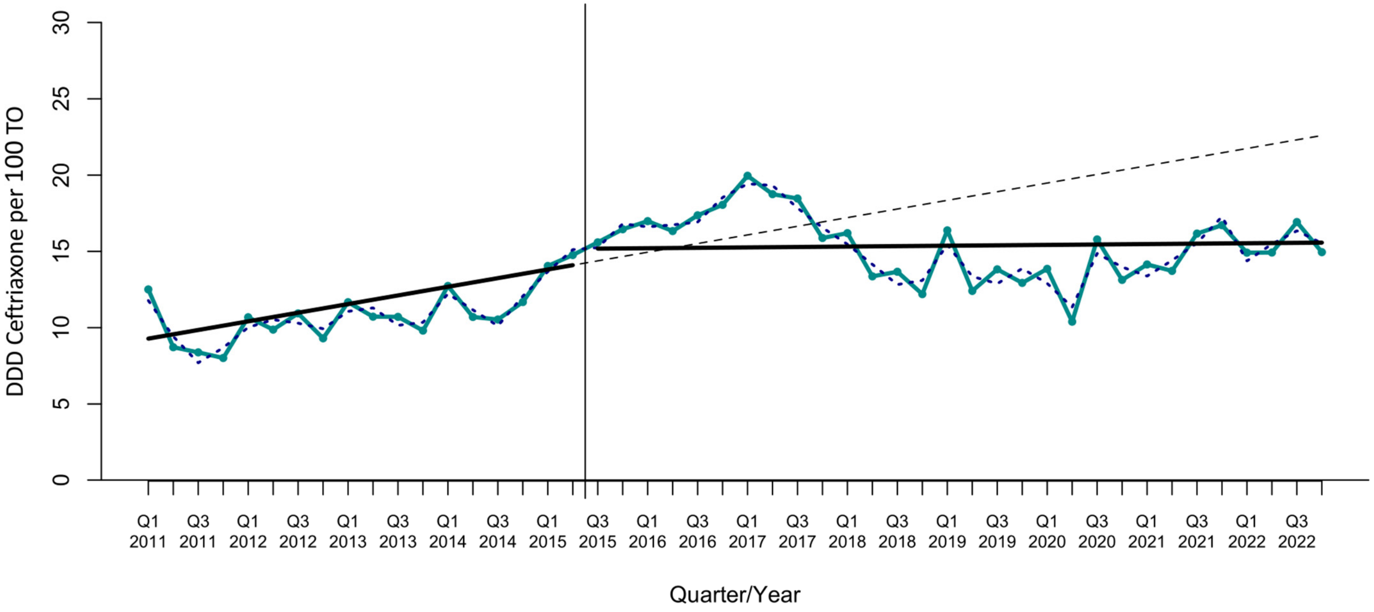

Figure S1. Interrupted time-series analysis of the trends in ceftriaxone observed before and after the implementation of the antimicrobial stewardship program.

Solid lines show the observed trend during the pre-intervention and intervention periods. Dashed line shows the expected trend after the intervention according to the pre-intervention values. The dotted blue line shows the deseasonalized series. DDDs, defined daily doses. TOs, patients transferred to the Observation Unit. Q, quarter.

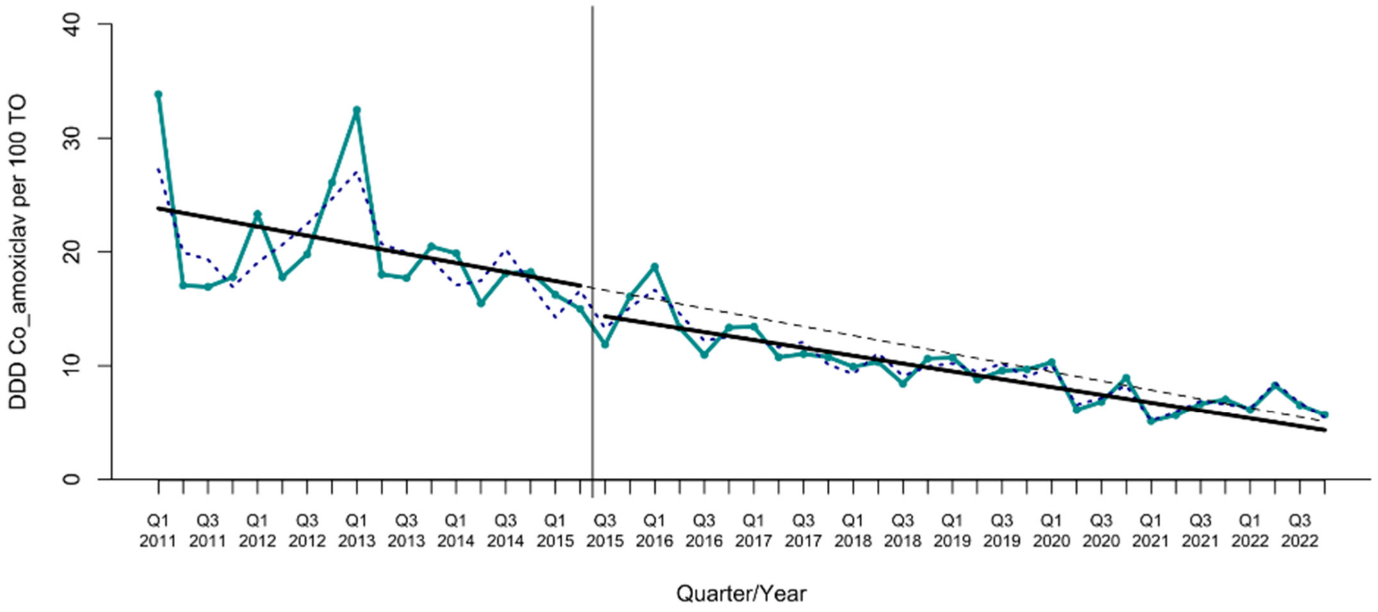

Figure S2. Interrupted time-series analysis of the trends in amoxicillin-clavulanic acid observed before and after the implementation of the antimicrobial stewardship program.

Solid lines show the observed trend during the pre-intervention and intervention periods. The dashed line shows the expected trend after the intervention according to the pre-intervention values. The dotted blue line shows the deseasonalized series. DDDs, defined daily doses. TOs, patients transferred to the Observation Unit. Q, quarter.

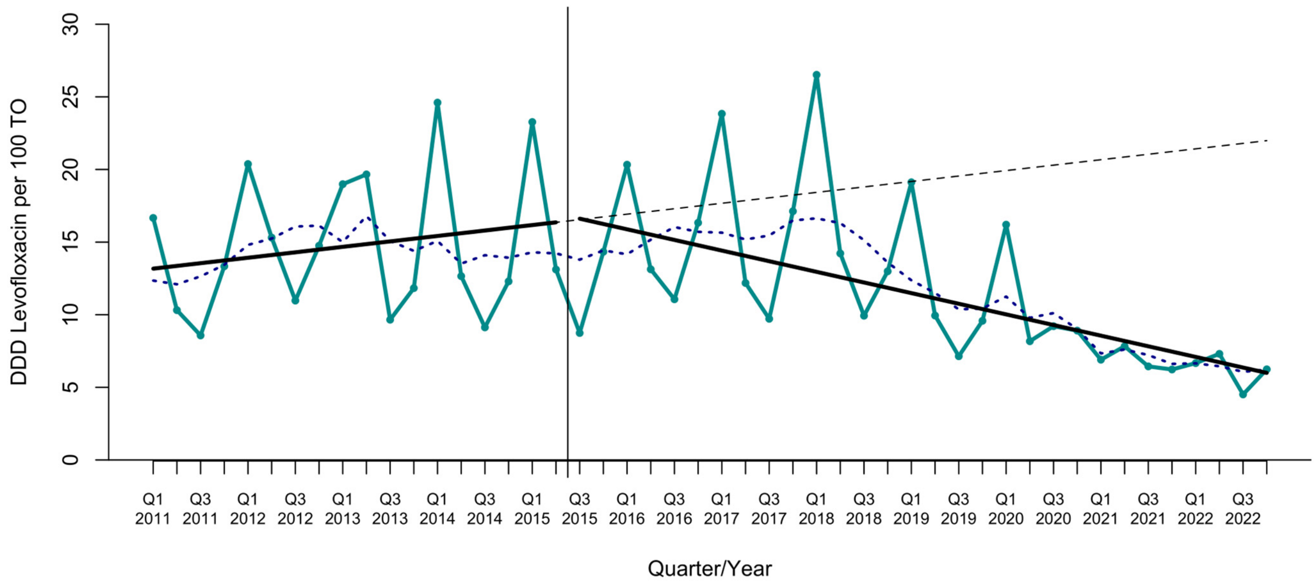

Figure S3. Interrupted time-series analysis of the trends in levofloxacin observed before and after the implementation of the antimicrobial stewardship program.

Solid lines show the observed trend during the pre-intervention and intervention periods. The dashed line shows the expected trend after the intervention according to the pre-intervention values. The dotted blue line shows the deseasonalized series. DDDs, defined daily doses. TOs, patients transferred to the Observation Unit. Q, quarter.

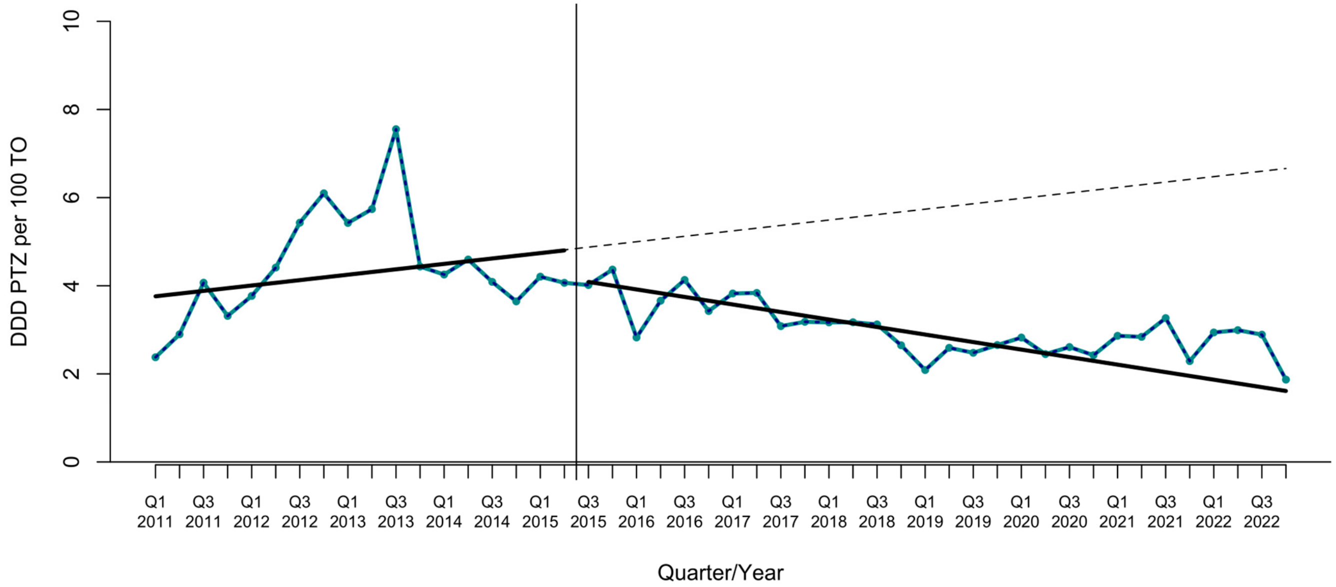

Figure S4. Interrupted time-series analysis of the trends in piperacillin-tazobactam (PTZ) observed before and after the implementation of the antimicrobial stewardship program.

Solid lines show the observed trend during the pre-intervention and intervention periods. The dashed line shows the expected trend after the intervention according to the pre-intervention values. DDDs, defined daily doses. TOs, patients transferred to the Observation Unit. Q, quarter.

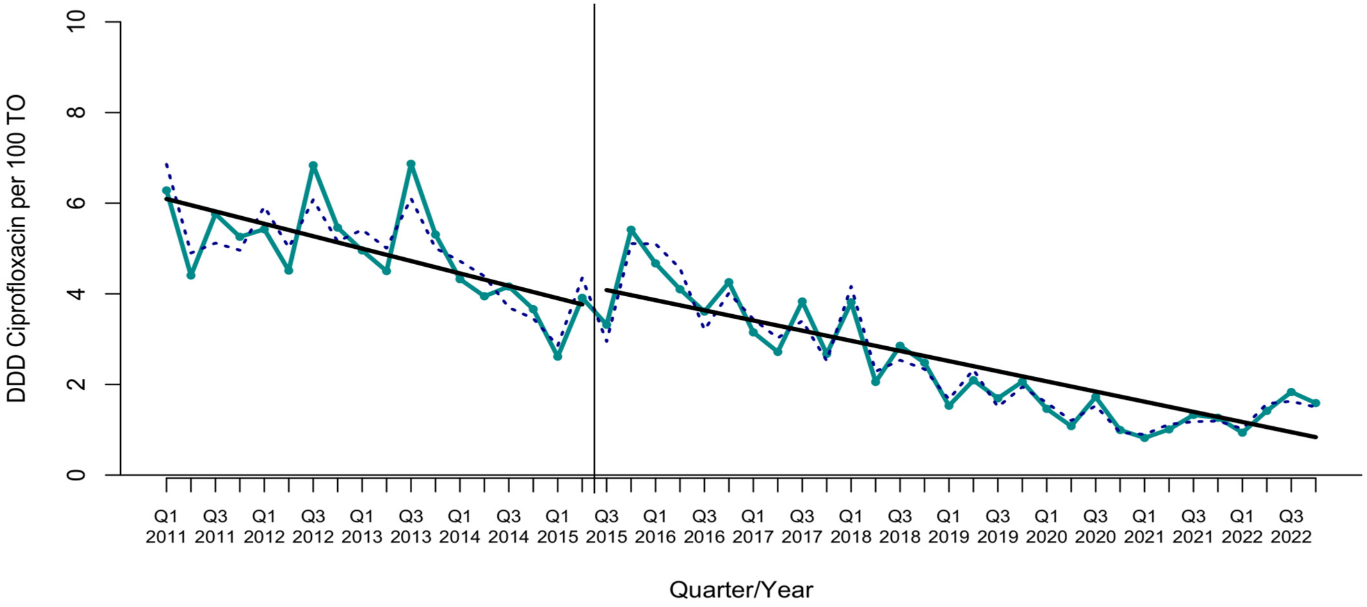

Figure S5. Interrupted time-series analysis of the trends in ciprofloxacin observed before and after the implementation of the antimicrobial stewardship program.

Solid lines show the observed trend during the pre-intervention and intervention periods. The dashed line shows the expected trend after the intervention according to the pre-intervention values. The dotted blue line shows the deseasonalized series. DDDs, defined daily doses. TOs, patients transferred to the Observation Unit. Q, quarter.

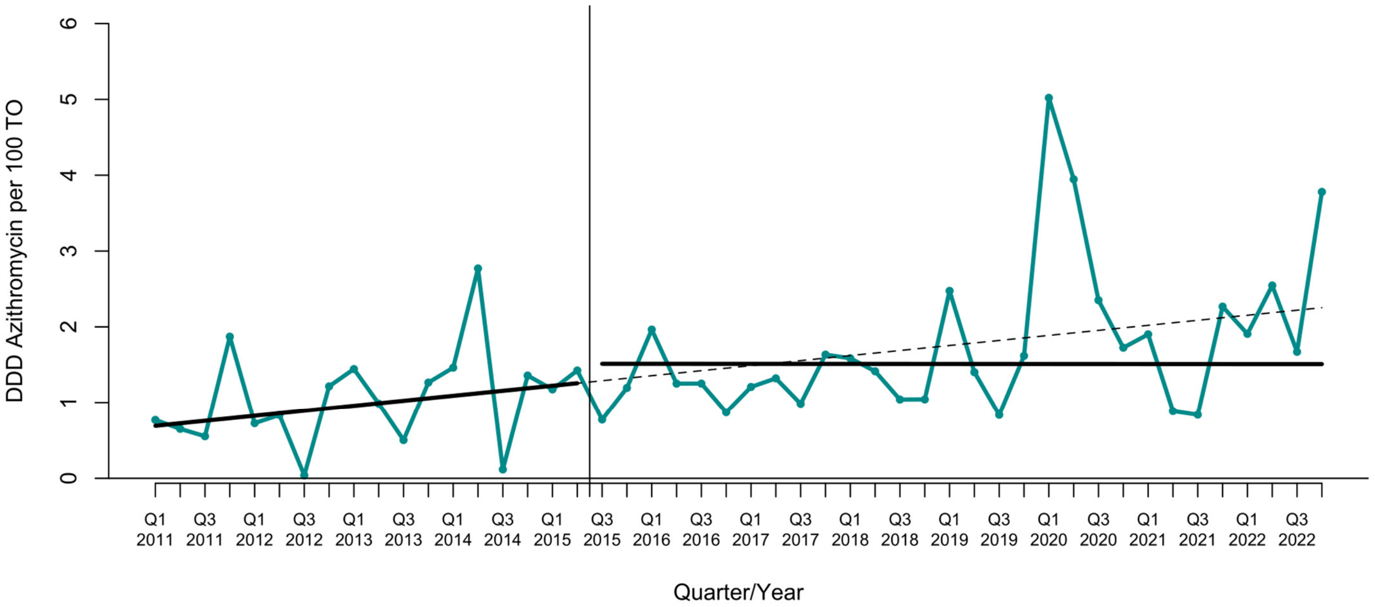

Figure S6. Interrupted time-series analysis of the trends in azithromycin observed before and after the implementation of the antimicrobial stewardship program.

Solid lines show the observed trend during the pre-intervention and intervention periods. The dashed line shows the expected trend after the intervention according to the pre-intervention values. DDDs, defined daily doses. TOs, patients transferred to the Observation Unit. Q, quarter.

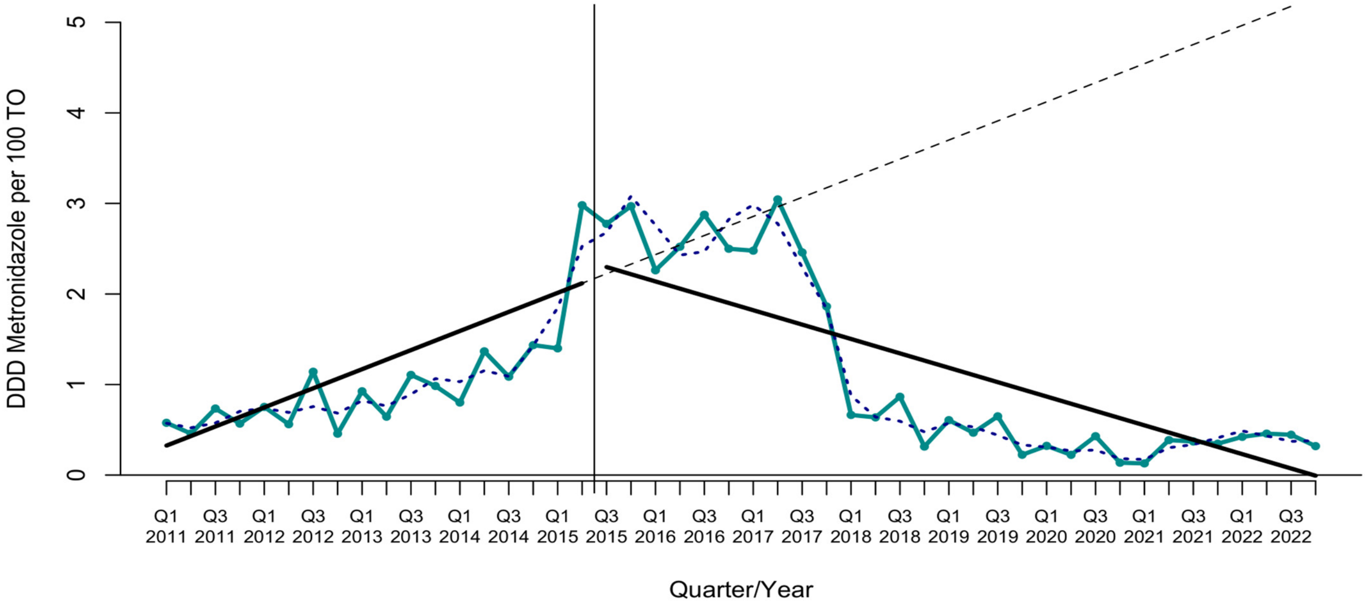

Figure S7. Interrupted time-series analysis of the trends in metronidazole observed before and after the implementation of the antimicrobial stewardship program.

Solid lines show the observed trend during the pre-intervention and intervention periods. The dashed line shows the expected trend after the intervention according to the pre-intervention values. The dotted blue line shows the deseasonalized series. DDDs, defined daily doses. TOs, patients transferred to the Observation Unit. Q, quarter.

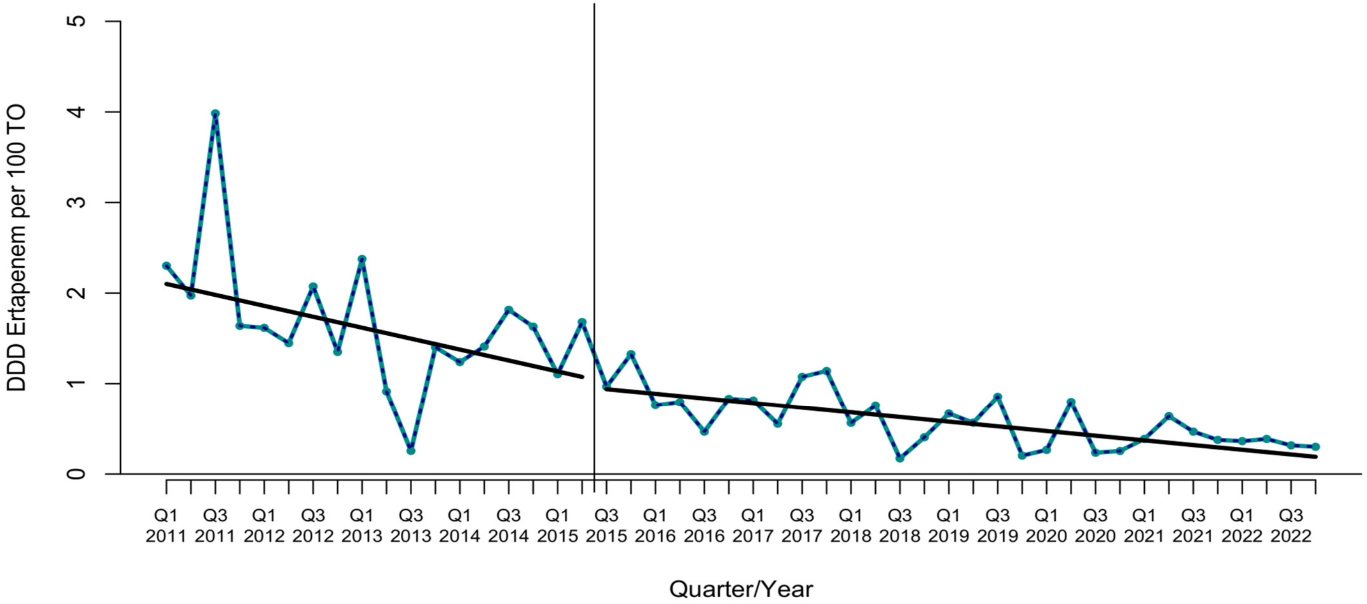

Figure S8. Interrupted time-series analysis of the trends in ertapenem observed before and after the implementation of the antimicrobial stewardship program.

Solid lines show the observed trend during the pre-intervention and intervention periods. DDDs, defined daily doses. TOs, patients transferred to the Observation Unit. Q, quarter.

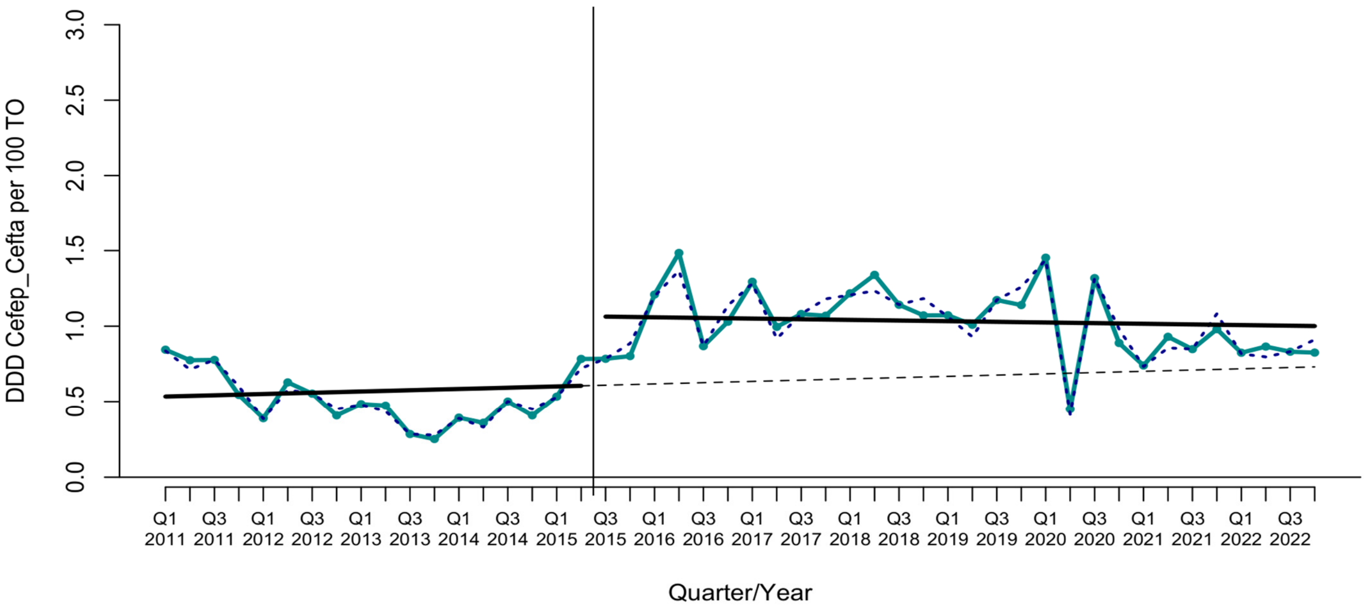

Figure S9. Interrupted time-series analysis of the trends in cefepime and ceftazidime observed before and after the implementation of the antimicrobial stewardship program.

Solid lines show the observed trend during the pre-intervention and intervention periods. The dashed line shows the expected trend after the intervention according to the pre-intervention values. The dotted blue line shows the deseasonalized series. DDDs, defined daily doses. TOs, patients transferred to the Observation Unit. Q, quarter.

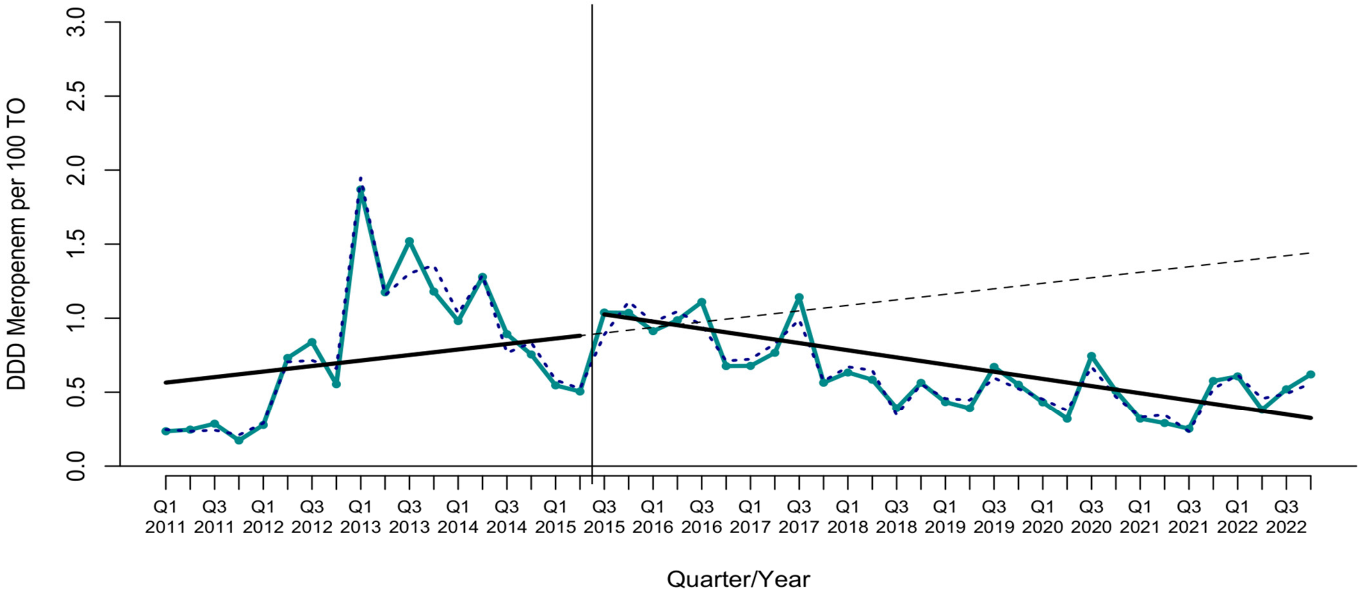

Figure S10. Interrupted time-series analysis of the trends in meropenem observed before and after the implementation of the antimicrobial stewardship program.

Solid lines show the observed trend during the pre-intervention and intervention periods. The dashed line shows the expected trend after the intervention according to the pre-intervention values. The dotted blue line shows the deseasonalized series. DDDs, defined daily doses. TOs, patients transferred to the Observation Unit. Q, quarter.

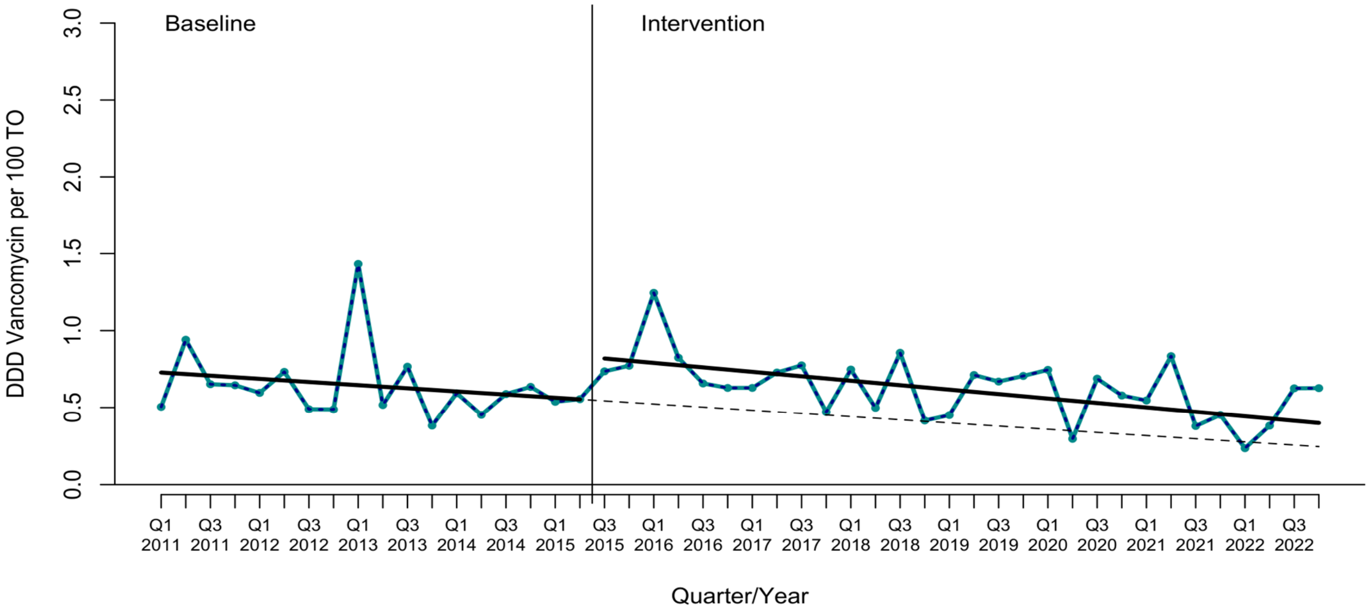

Figure S11. Interrupted time-series analysis of the trends in vancomycin observed before and after the implementation of the antimicrobial stewardship program.

Solid lines show the observed trend during the pre-intervention and intervention periods. The dashed line shows the expected trend after the intervention according to the pre-intervention values. DDDs, defined daily doses. TOs, patients transferred to the Observation Unit. Q, quarter.

Table S2. Data of the blood cultures collected annually in the Emergency Department (ED) per 100 ED visits.

Year	Number of Blood Cultures	ED visits	Blood Cultures per 100 ED Visits
2011	16,086	119,389	13
2012	16,210	116,128	14
2013	16,724	115,721	14
2014	16,754	116,139	14
2015	17,903	119,837	15
2016	18,621	125,410	15
2017	19,980	125,520	16
2018	20,300	125,011	16
2019	22,849	133,467	17
2020	19,466	104,258	19
2021	22,153	123,224	18
2022	23,830	132,560	18

Table S3. Incidence of bloodstream infections and mortality of patients diagnosed with bloodstream infections by causative microorganisms (2011–2022).

Microorganism	Number of BSI	Number of Deaths on Day +14 (%)
Overall	3392	317 (9.3)
Escherichia coli	2043	151 (7.4)
ESBL *E. coli*	195	16 (8.2)
Klebsiella pneumoniae	530	40 (7.5)
ESBL *K. pneumoniae*	81	10 (12.3)
Pseudomonas aeruginosa	250	39 (15.6)
MDR *P. aeruginosa*	15	2 (13.3)
Staphylococcus aureus	370	55 (14.9)
MRSA	41	9 (22.0)
Streptococcus pneumoniae	199	32 (16.1)

ESBL, extended-spectrum β-lactamase; MDR, multidrug-resistant; MRSA, methicillin-resistant *Staphylococcus aureus*; BSIs, bloodstream infections.

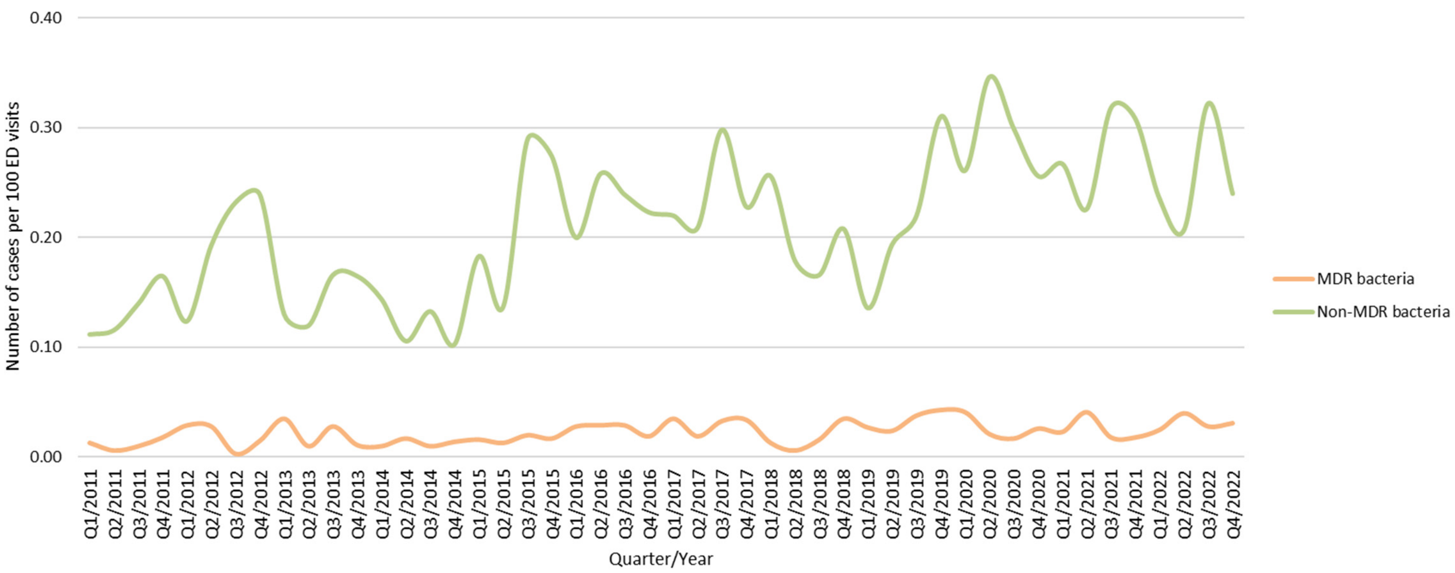

Figure S12. Time-series plot of incidence density of bloodstream infections during the study period (2011–2022). Data are presented as the quarterly number of cases per 100 Emergency Department (ED) visits.

"MDR bacteria" refer to extended-spectrum β-lactamase (ESBL) *E. coli*, ESBL *K. pneumoniae*, MDR *P. aeruginosa*, and methicillin-resistant *S. aureus*. "Non-MDR bacteria" include all other bloodstream infection pathogens monitored.

Table S4. Differences between the pre-intervention period and the antimicrobial stewardship program (ASP) period regarding the pre-post analysis of incidence density of bloodstream infections.

**Outcomes**	**Pre-Intervention Period**	**ASP Period**	***p*-value**
**Overall BSIs**	0.17±0.04	0.27±0.05	<0.001
MDR bacteria	0.02±0.01	0.03±0.01	<0.001
Non-MDR bacteria	0.15±0.04	0.25±0.05	<0.001

Data are presented as mean ± standard deviation of the quarterly number of cases per 100 Emergency Department visits. *p*-values represent the results from Student's t-tests or Mann–Whitney U tests, according to the data distribution. "MDR bacteria" refer to extended-spectrum β-lactamase (ESBL) *E. coli*, ESBL *K. pneumoniae*, MDR *P. aeruginosa*, and methicillin-resistant *S. aureus*. "Non-MDR bacteria" include all other bloodstream infection pathogens monitored. BSIs, bloodstream infections.

Table S5. Interrupted time-series analysis of changes in trends of the incidence density of bloodstream infections.

Outcomes	Regression Intercept	Pre-Intervention Trend	Change in Level [a]	Change in Trend [b]	Absolute Effect	Relative Effect [c] (%)
Overall BSI	0.171 (0.111 to 0.230)	−0.001 (−0.006 to 0.005)	0.099 (0.032 to 0.167)	0.002 (−0.004 to 0.008)	0.161 (0.054 to 0.268)	123.2 (41.3 to 284.7)
MDR bacteria	0.016 (0.007 to 0.026)	−0.0001 (−0.001 to 0.001)	0.008 (−0.003 to 0.019)	0.0002 (−0.001 to 0.001)	0.015 (−0.018 to 0.049)	111.7 (−402.2 to 625.5)
Non-MDR bacteria	0.155 (0.098 to 0.211)	−0.001 (−0.006 to 0.004)	0.088 (0.023 to 0.154)	0.002 (−0.004 to 0.008)	0.143 (0.004 to 0.282)	119.9 (23.9 to 215.7)

Data are presented as the quarterly number of cases per 100 Emergency Department visits with a 95% confidence interval unless otherwise specified. [a] Increase or decrease in the first quarter after the start of the antimicrobial stewardship program (ASP) period with respect to the expected value. [b] Change in slope for the ASP period. [c] Percentage difference between the expected value according to the pre-intervention trend and the trend at the end of the ASP period. "MDR bacteria" refer to extended-spectrum β-lactamase (ESBL) *E. coli*, ESBL *K. pneumoniae*, MDR *P. aeruginosa*, and methicillin-resistant *S. aureus*. "Non-MDR bacteria" include all other bloodstream infection pathogens monitored. BSIs, bloodstream infections.

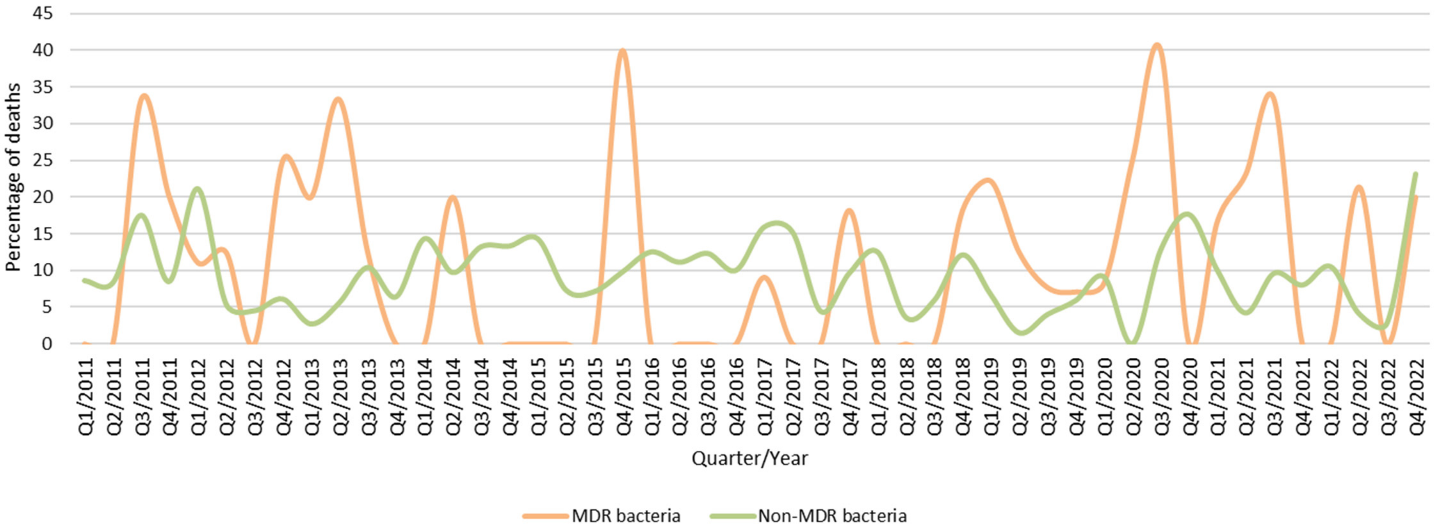

Figure S13. Time-series plot of mortality rate of bloodstream infections during the study period (2011–2022). Data are presented as quarterly percentage of all-cause deaths on day +14.

"MDR bacteria" refer to extended-spectrum β-lactamase (ESBL) *E. coli*, ESBL *K. pneumoniae*, MDR *P. aeruginosa*, and methicillin-resistant *S. aureus*. "Non-MDR bacteria" include all other bloodstream infection pathogens monitored.

Table S6. Differences between the pre-intervention period and the antimicrobial stewardship program (ASP) period regarding the pre-post analysis of the mortality rate of bloodstream infections.

**Outcomes**	**Pre-Intervention Period**	**ASP Period**	***p*-value**
**Overall BSIs**	9.98 ± 4.17	9.25 ± 4.64	0.587
MDR bacteria	10.43 ± 12.18	10.76 ± 12.74	0.946
Non-MDR bacteria	9.84 ± 4.87	9.07 ± 5.10	0.606

Data are presented as the mean ± standard deviation of the quarterly percentage of all-cause deaths on day +14. *p*-values represent the results from Student's t-tests or Mann–Whitney U tests, according to the data distribution. "MDR bacteria" refer to extended-spectrum β-lactamase (ESBL) *E. coli*, ESBL *K. pneumoniae*, MDR *P. aeruginosa*, and methicillin-resistant *S. aureus*. "Non-MDR bacteria" include all other bloodstream infection pathogens monitored. BSIs, bloodstream infections.

Table S7. Interrupted time-series analysis of changes in trends of the mortality rate of bloodstream infections.

Outcomes	Regression Intercept	Pre-Intervention Trend	Change in Level [a]	Change in Trend [b]	Absolute Effect
Overall BSIs	10.03 (5.85 to 14.22)	−0.004 (−0.39 to 0.38)	−0.66 (−5.67 to 4.35)	−0.001 (−0.43 to 0.43)	−0.67 (−15.99 to 14.65)
MDR bacteria	17.19 (5.42 to 28.96)	−0.71 (−1.80 to 0.38)	0.59 (−13.48 to 14.65)	1.09 (−0.12 to 2.28)	16.70 (−5.05 to 38.45)
Non-MDR bacteria	9.33 (4.29 to 14.38)	0.05 (−0.41 to 0.51)	−0.49 (−6.50 to 5.52)	−0.10 (−0.61 to 0.42)	−3.42 (−21.88 to 15.03)

Data are presented as a quarterly percentage of deaths on day +14 with a 95% confidence interval unless otherwise specified. [a] Increase or decrease in the first quarter after the start of the antimicrobial stewardship program (ASP) period with respect to the expected value. [b] Change in slope for the ASP period. "MDR bacteria" refer to extended-spectrum β-lactamase (ESBL) *E. coli*, ESBL *K. pneumoniae*, MDR *P. aeruginosa*, and methicillin-resistant *S. aureus*. "Non-MDR bacteria" include all other bloodstream infection pathogens monitored. BSIs, bloodstream infections.
